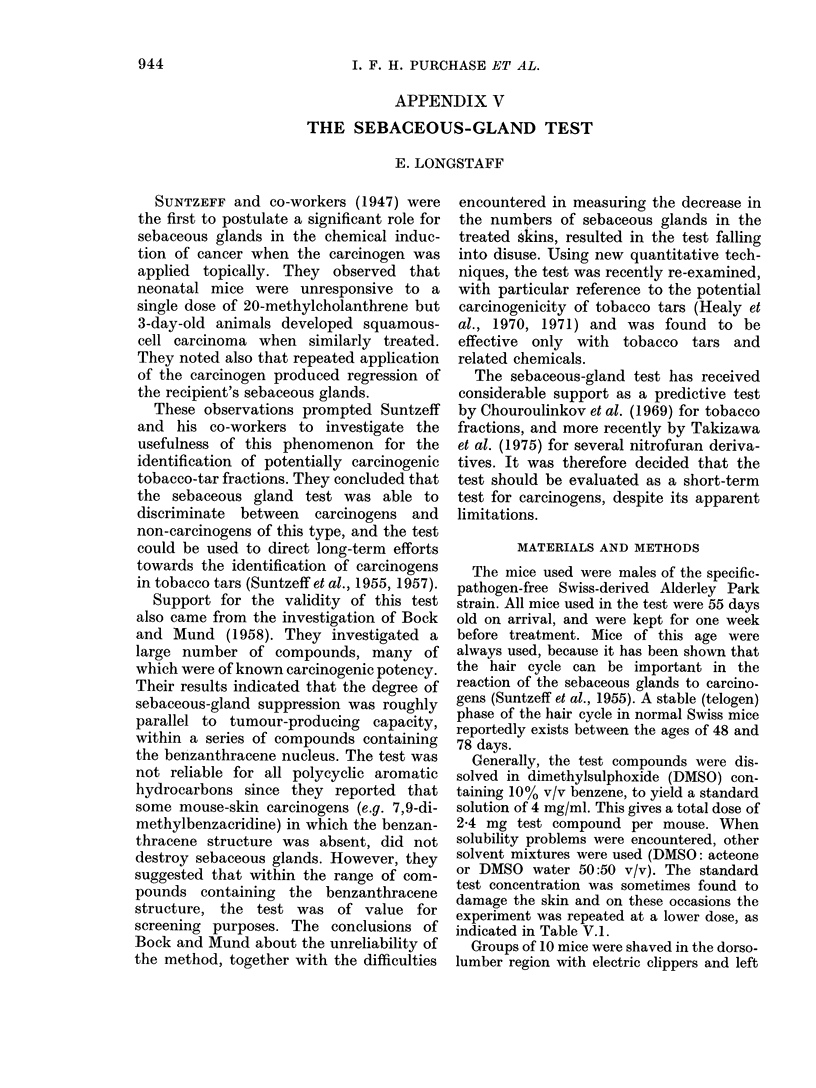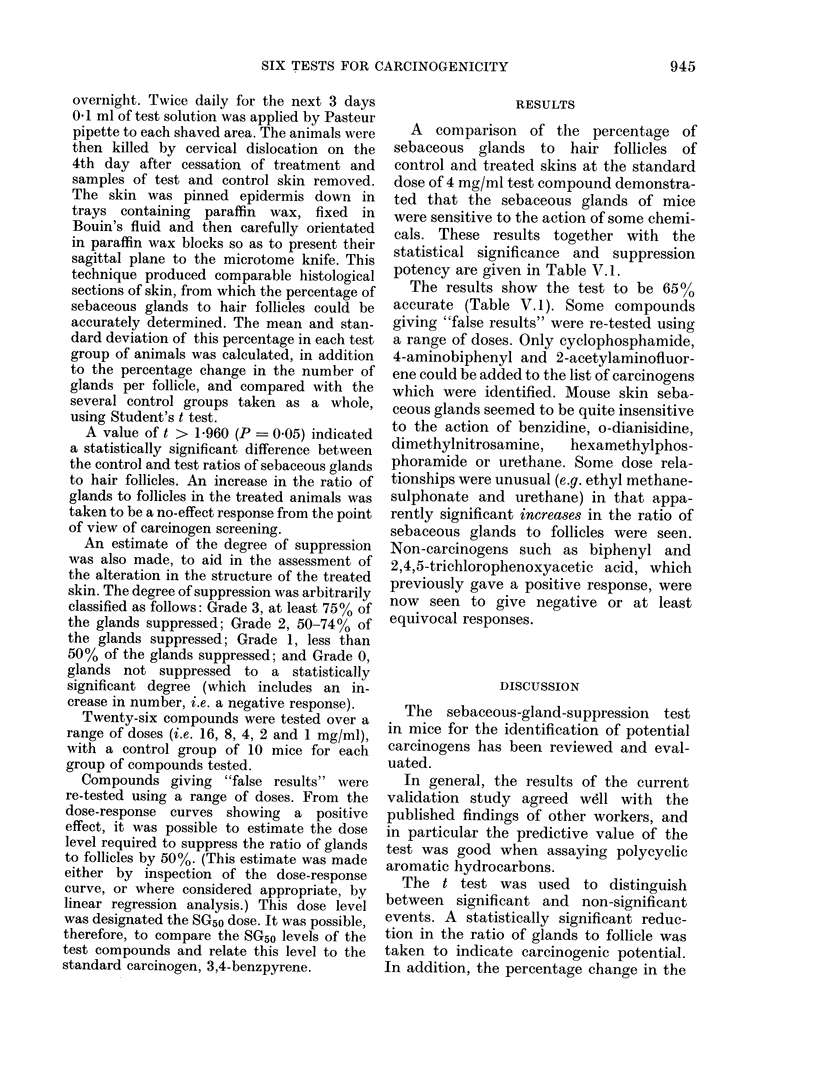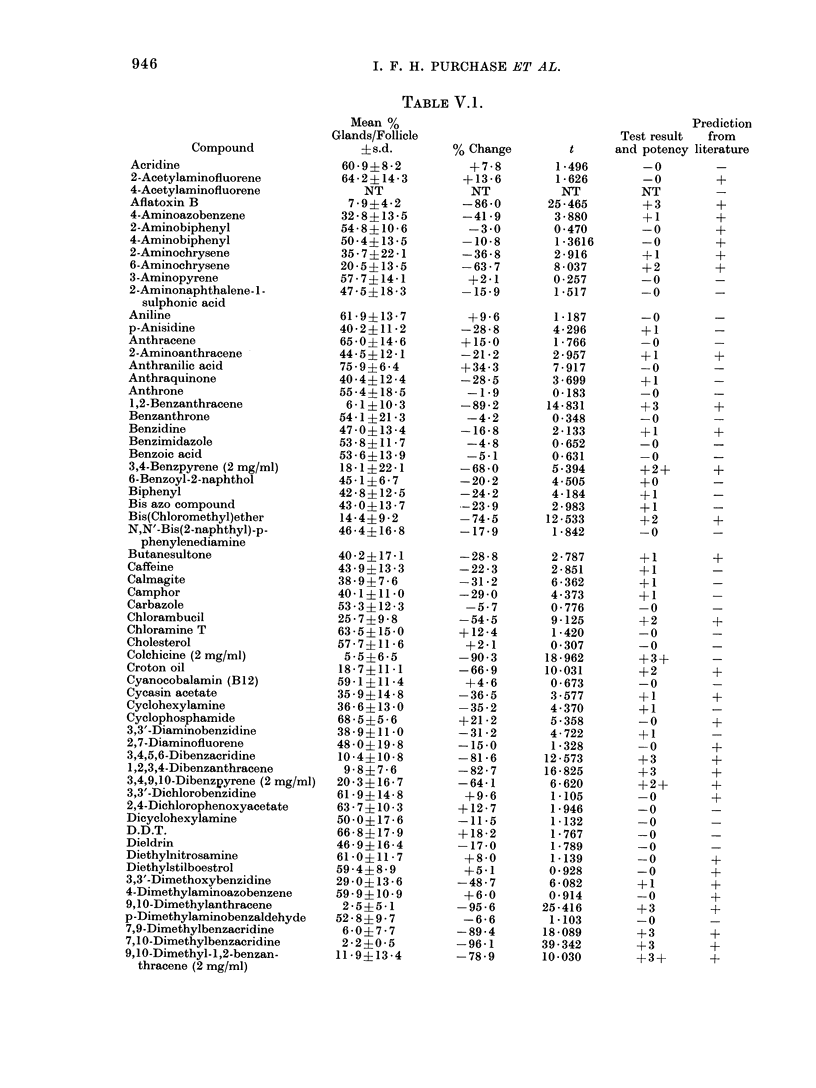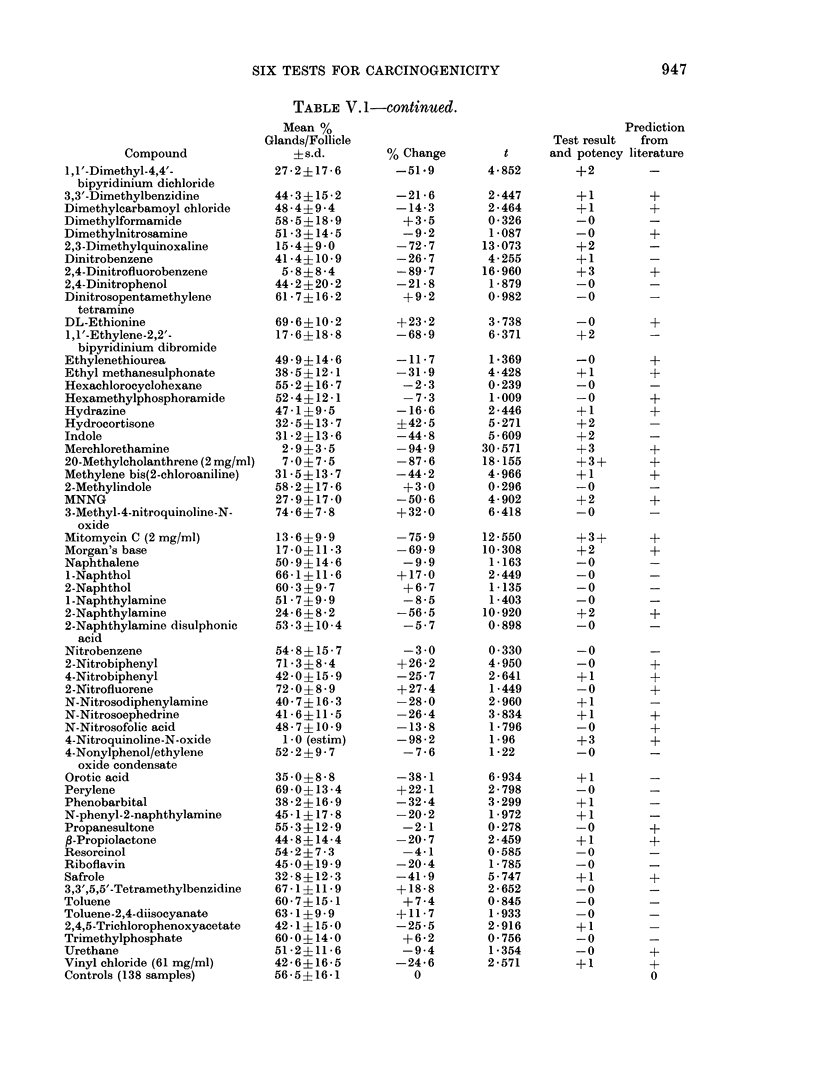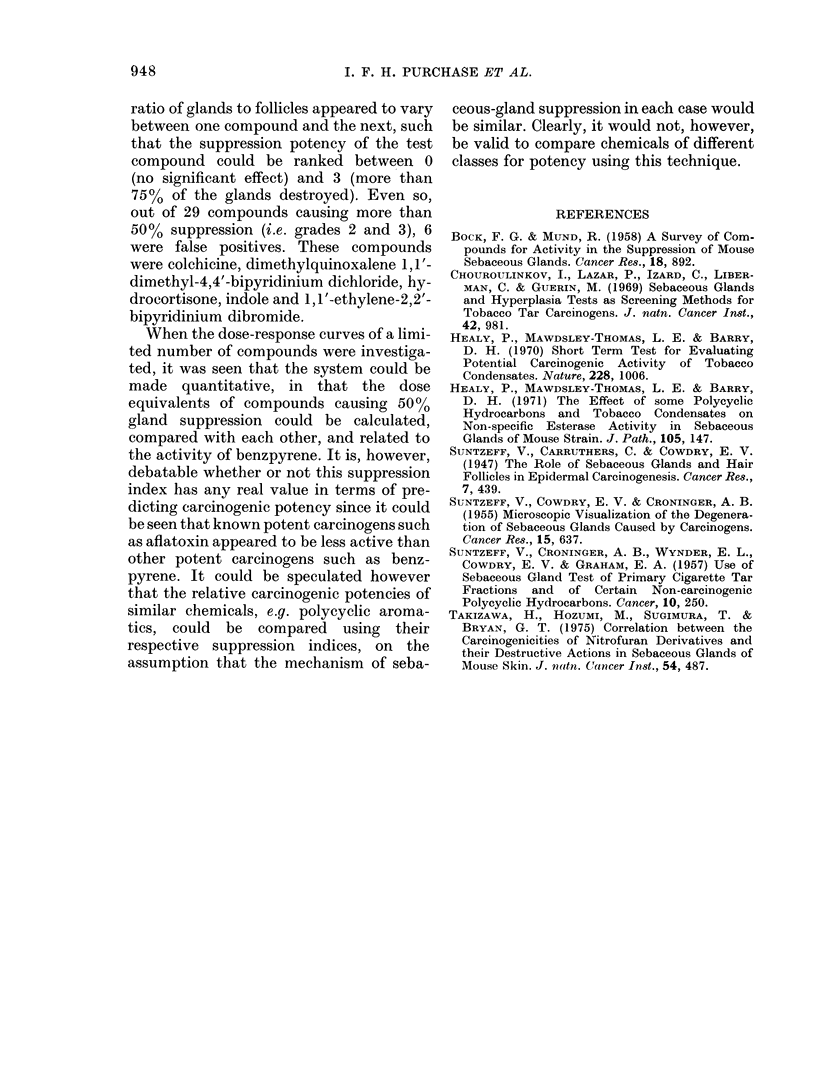# The sebaceous-gland test. Six tests for carcinogenicity.

**DOI:** 10.1038/bjc.1978.137

**Published:** 1978-06

**Authors:** E. Longstaff


### APPENDIX V

### THE SEBACEOUS-GLAND TEST

E. LONGSTAFF

SUNTZEFF and co-workers (1947) were the first to postulate a significant role for sebaceous glands in the chemical induction of cancer when the carcinogen was applied topically. They observed that neonatal mice were unresponsive to a single dose of 20-methylcholanthrene but 3-day-old animals developed squamous-cell carcinoma when similarly treated. They noted also that repeated application of the carcinogen produced regression of the recipient's sebaceous glands.

These observations prompted Suntzeff and his co-workers to investigate the usefulness of this phenomenon for the identification of potentially carcinogenic tobacco-tar fractions. They concluded that the sebaceous gland test was able to discriminate between carcinogens and non-carcinogens of this type, and the test could be used to direct long-term efforts towards the identification of carcinogens in tobacco tars (Suntzeff *et al.*, 1955, 1957).

Support for the validity of this test also came from the investigation of Bock and Mund (1958). They investigated a large number of compounds, many of which were of known carcinogenic potency. Their results indicated that the degree of sebaceous-gland suppression was roughly parallel to tumour-producing capacity, within a series of compounds containing the benzanthracene nucleus. The test was not reliable for all polycyclic aromatic hydrocarbons since they reported that some mouse-skin carcinogens (*e.g.* 7,9-dimethylbenzacridine) in which the benzanthracene structure was absent, did not destroy sebaceous glands. However, they suggested that within the range of compounds containing the benzanthracene structure, the test was of value for screening purposes. The conclusions of Bock and Mund about the unreliability of the method, together with the difficulties encountered in measuring the decrease in the numbers of sebaceous glands in the treated skins, resulted in the test falling into disuse. Using new quantitative techniques, the test was recently re-examined, with particular reference to the potential carcinogenicity of tobacco tars (Healy *et al.*, 1970, 1971) and was found to be effective only with tobacco tars and related chemicals.

The sebaceous-gland test has received considerable support as a predictive test by Chouroulinkov *et al.* (1969) for tobacco fractions, and more recently by Takizawa *et al.* (1975) for several nitrofuran derivatives. It was therefore decided that the test should be evaluated as a short-term test for carcinogens, despite its apparent limitations.

### MATERIALS AND METHODS

The mice used were males of the specific-pathogen-free Swiss-derived Alderley Park strain. All mice used in the test were 55 days old on arrival, and were kept for one week before treatment. Mice of this age were always used, because it has been shown that the hair cycle can be important in the reaction of the sebaceous glands to carcinogens (Suntzeff *et al.*, 1955). A stable (telogen) phase of the hair cycle in normal Swiss mice reportedly exists between the ages of 48 and 78 days.

Generally, the test compounds were dissolved in dimethylsulphoxide (DMSO) containing 10% v/v benzene, to yield a standard solution of 4 mg/ml. This gives a total dose of 2·4 mg test compound per mouse. When solubility problems were encountered, other solvent mixtures were used (DMSO: acteone or DMSO water 50:50 v/v). The standard test concentration was sometimes found to damage the skin and on these occasions the experiment was repeated at a lower dose, as indicated in Table V.1.

Groups of 10 mice were shaved in the dorso-lumber region with electric clippers and left overnight. Twice daily for the next 3 days 0·1 ml of test solution was applied by Pasteur pipette to each shaved area. The animals were then killed by cervical dislocation on the 4th day after cessation of treatment and samples of test and control skin removed. The skin was pinned epidermis down in trays containing paraffin wax, fixed in Bouin's fluid and then carefully orientated in paraffin wax blocks so as to present their sagittal plane to the microtome knife. This technique produced comparable histological sections of skin, from which the percentage of sebaceous glands to hair follicles could be accurately determined. The mean and standard deviation of this percentage in each test group of animals was calculated, in addition to the percentage change in the number of glands per follicle, and compared with the several control groups taken as a whole, using Student's *t* test.

A value of $t > 1{\cdot}960$ ($P = 0{\cdot}05$) indicated a statistically significant difference between the control and test ratios of sebaceous glands to hair follicles. An increase in the ratio of glands to follicles in the treated animals was taken to be a no-effect response from the point of view of carcinogen screening.

An estimate of the degree of suppression was also made, to aid in the assessment of the alteration in the structure of the treated skin. The degree of suppression was arbitrarily classified as follows: Grade 3, at least 75% of the glands suppressed; Grade 2, 50–74% of the glands suppressed; Grade 1, less than 50% of the glands suppressed; and Grade 0, glands not suppressed to a statistically significant degree (which includes an increase in number, *i.e.* a negative response).

Twenty-six compounds were tested over a range of doses (*i.e.* 16, 8, 4, 2 and 1 mg/ml), with a control group of 10 mice for each group of compounds tested.

Compounds giving "false results" were re-tested using a range of doses. From the dose-response curves showing a positive effect, it was possible to estimate the dose level required to suppress the ratio of glands to follicles by 50%. (This estimate was made either by inspection of the dose-response curve, or where considered appropriate, by linear regression analysis.) This dose level was designated the $SG_{50}$ dose. It was possible, therefore, to compare the $SG_{50}$ levels of the test compounds and relate this level to the standard carcinogen, 3,4-benzpyrene.

### RESULTS

A comparison of the percentage of sebaceous glands to hair follicles of control and treated skins at the standard dose of 4 mg/ml test compound demonstrated that the sebaceous glands of mice were sensitive to the action of some chemicals. These results together with the statistical significance and suppression potency are given in Table V.1.

The results show the test to be 65% accurate (Table V.1). Some compounds giving "false results" were re-tested using a range of doses. Only cyclophosphamide, 4-aminobiphenyl and 2-acetylaminofluorene could be added to the list of carcinogens which were identified. Mouse skin sebaceous glands seemed to be quite insensitive to the action of benzidine, o-dianisidine, dimethylnitrosamine, hexamethylphosphoramide or urethane. Some dose relationships were unusual (*e.g.* ethyl methanesulphonate and urethane) in that apparently significant *increases* in the ratio of sebaceous glands to follicles were seen. Non-carcinogens such as biphenyl and 2,4,5-trichlorophenoxyacetic acid, which previously gave a positive response, were now seen to give negative or at least equivocal responses.

### DISCUSSION

The sebaceous-gland-suppression test in mice for the identification of potential carcinogens has been reviewed and evaluated.

In general, the results of the current validation study agreed well with the published findings of other workers, and in particular the predictive value of the test was good when assaying polycyclic aromatic hydrocarbons.

The *t* test was used to distinguish between significant and non-significant events. A statistically significant reduction in the ratio of glands to follicle was taken to indicate carcinogenic potential. In addition, the percentage change in the

TABLE V.1.

| Compound | Mean % Glands/Follicle ±s.d. | % Change | $t$ | Test result and potency | Prediction from literature |
|---|---|---|---|---|---|
| Acridine | 60·9±8·2 | +7·8 | 1·496 | −0 | − |
| 2-Acetylaminofluorene | 64·2±14·3 | +13·6 | 1·626 | −0 | + |
| 4-Acetylaminofluorene | NT | NT | NT | NT | − |
| Aflatoxin B | 7·9±4·2 | −86·0 | 25·465 | +3 | + |
| 4-Aminoazobenzene | 32·8±13·5 | −41·9 | 3·880 | +1 | + |
| 2-Aminobiphenyl | 54·8±10·6 | −3·0 | 0·470 | −0 | + |
| 4-Aminobiphenyl | 50·4±13·5 | −10·8 | 1·3616 | −0 | + |
| 2-Aminochrysene | 35·7±22·1 | −36·8 | 2·916 | +1 | + |
| 6-Aminochrysene | 20·5±13·5 | −63·7 | 8·037 | +2 | + |
| 3-Aminopyrene | 57·7±14·1 | +2·1 | 0·257 | −0 | − |
| 2-Aminonaphthalene-1-sulphonic acid | 47·5±18·3 | −15·9 | 1·517 | −0 | − |
| Aniline | 61·9±13·7 | +9·6 | 1·187 | −0 | − |
| p-Anisidine | 40·2±11·2 | −28·8 | 4·296 | +1 | − |
| Anthracene | 65·0±14·6 | +15·0 | 1·766 | −0 | − |
| 2-Aminoanthracene | 44·5±12·1 | −21·2 | 2·957 | +1 | + |
| Anthranilic acid | 75·9±6·4 | +34·3 | 7·917 | −0 | − |
| Anthraquinone | 40·4±12·4 | −28·5 | 3·699 | +1 | − |
| Anthrone | 55·4±18·5 | −1·9 | 0·183 | −0 | − |
| 1,2-Benzanthracene | 6·1±10·3 | −89·2 | 14·831 | +3 | + |
| Benzanthrone | 54·1±21·3 | −4·2 | 0·348 | −0 | − |
| Benzidine | 47·0±13·4 | −16·8 | 2·133 | +1 | + |
| Benzimidazole | 53·8±11·7 | −4·8 | 0·652 | −0 | − |
| Benzoic acid | 53·6±13·9 | −5·1 | 0·631 | −0 | − |
| 3,4-Benzpyrene (2 mg/ml) | 18·1±22·1 | −68·0 | 5·394 | +2+ | + |
| 6-Benzoyl-2-naphthol | 45·1±6·7 | −20·2 | 4·505 | +0 | − |
| Biphenyl | 42·8±12·5 | −24·2 | 4·184 | +1 | − |
| Bis azo compound | 43·0±13·7 | −23·9 | 2·983 | +1 | − |
| Bis(Chloromethyl)ether | 14·4±9·2 | −74·5 | 12·533 | +2 | + |
| N,N′-Bis(2-naphthyl)-p-phenylenediamine | 46·4±16·8 | −17·9 | 1·842 | −0 | − |
| Butanesultone | 40·2±17·1 | −28·8 | 2·787 | +1 | + |
| Caffeine | 43·9±13·3 | −22·3 | 2·851 | +1 | − |
| Calmagite | 38·9±7·6 | −31·2 | 6·362 | +1 | − |
| Camphor | 40·1±11·0 | −29·0 | 4·373 | +1 | − |
| Carbazole | 53·3±12·3 | −5·7 | 0·776 | −0 | − |
| Chlorambucil | 25·7±9·8 | −54·5 | 9·125 | +2 | + |
| Chloramine T | 63·5±15·0 | +12·4 | 1·420 | −0 | − |
| Cholesterol | 57·7±11·6 | +2·1 | 0·307 | −0 | − |
| Colchicine (2 mg/ml) | 5·5±6·5 | −90·3 | 18·962 | +3+ | − |
| Croton oil | 18·7±11·1 | −66·9 | 10·031 | +2 | + |
| Cyanocobalamin (B12) | 59·1±11·4 | +4·6 | 0·673 | −0 | − |
| Cycasin acetate | 35·9±14·8 | −36·5 | 3·577 | +1 | + |
| Cyclohexylamine | 36·6±13·0 | −35·2 | 4·370 | +1 | − |
| Cyclophosphamide | 68·5±5·6 | +21·2 | 5·358 | −0 | + |
| 3,3′-Diaminobenzidine | 38·9±11·0 | −31·2 | 4·722 | +1 | − |
| 2,7-Diaminofluorene | 48·0±19·8 | −15·0 | 1·328 | −0 | + |
| 3,4,5,6-Dibenzacridine | 10·4±10·8 | −81·6 | 12·573 | +3 | + |
| 1,2,3,4-Dibenzanthracene | 9·8±7·6 | −82·7 | 16·825 | +3 | + |
| 3,4,9,10-Dibenzpyrene (2 mg/ml) | 20·3±16·7 | −64·1 | 6·620 | +2+ | + |
| 3,3′-Dichlorobenzidine | 61·9±14·8 | +9·6 | 1·105 | −0 | + |
| 2,4-Dichlorophenoxyacetate | 63·7±10·3 | +12·7 | 1·946 | −0 | − |
| Dicyclohexylamine | 50·0±17·6 | −11·5 | 1·132 | −0 | − |
| D.D.T. | 66·8±17·9 | +18·2 | 1·767 | −0 | − |
| Dieldrin | 46·9±16·4 | −17·0 | 1·789 | −0 | − |
| Diethylnitrosamine | 61·0±11·7 | +8·0 | 1·139 | −0 | + |
| Diethylstilboestrol | 59·4±8·9 | +5·1 | 0·928 | −0 | + |
| 3,3′-Dimethoxybenzidine | 29·0±13·6 | −48·7 | 6·082 | +1 | + |
| 4-Dimethylaminoazobenzene | 59·9±10·9 | +6·0 | 0·914 | −0 | + |
| 9,10-Dimethylanthracene | 2·5±5·1 | −95·6 | 25·416 | +3 | + |
| p-Dimethylaminobenzaldehyde | 52·8±9·7 | −6·6 | 1·103 | −0 | − |
| 7,9-Dimethylbenzacridine | 6·0±7·7 | −89·4 | 18·089 | +3 | + |
| 7,10-Dimethylbenzacridine | 2·2±0·5 | −96·1 | 39·342 | +3 | + |
| 9,10-Dimethyl-1,2-benzanthracene (2 mg/ml) | 11·9±13·4 | −78·9 | 10·030 | +3+ | + |

TABLE V.1—*continued.*

| Compound | Mean % Glands/Follicle ±s.d. | % Change | *t* | Test result and potency | Prediction from literature |
|---|---|---|---|---|---|
| 1,1′-Dimethyl-4,4′-bipyridinium dichloride | 27·2±17·6 | −51·9 | 4·852 | +2 | − |
| 3,3′-Dimethylbenzidine | 44·3±15·2 | −21·6 | 2·447 | +1 | + |
| Dimethylcarbamoyl chloride | 48·4±9·4 | −14·3 | 2·464 | +1 | + |
| Dimethylformamide | 58·5±18·9 | +3·5 | 0·326 | −0 | − |
| Dimethylnitrosamine | 51·3±14·5 | −9·2 | 1·087 | −0 | + |
| 2,3-Dimethylquinoxaline | 15·4±9·0 | −72·7 | 13·073 | +2 | − |
| Dinitrobenzene | 41·4±10·9 | −26·7 | 4·255 | +1 | − |
| 2,4-Dinitrofluorobenzene | 5·8±8·4 | −89·7 | 16·960 | +3 | + |
| 2,4-Dinitrophenol | 44·2±20·2 | −21·8 | 1·879 | −0 | − |
| Dinitrosopentamethylene tetramine | 61·7±16·2 | +9·2 | 0·982 | −0 | − |
| DL-Ethionine | 69·6±10·2 | +23·2 | 3·738 | −0 | + |
| 1,1′-Ethylene-2,2′-bipyridinium dibromide | 17·6±18·8 | −68·9 | 6·371 | +2 | − |
| Ethylenethiourea | 49·9±14·6 | −11·7 | 1·369 | −0 | + |
| Ethyl methanesulphonate | 38·5±12·1 | −31·9 | 4·428 | +1 | + |
| Hexachlorocyclohexane | 55·2±16·7 | −2·3 | 0·239 | −0 | − |
| Hexamethylphosphoramide | 52·4±12·1 | −7·3 | 1·009 | −0 | + |
| Hydrazine | 47·1±9·5 | −16·6 | 2·446 | +1 | + |
| Hydrocortisone | 32·5±13·7 | ±42·5 | 5·271 | +2 | − |
| Indole | 31·2±13·6 | −44·8 | 5·609 | +2 | − |
| Merchlorethamine | 2·9±3·5 | −94·9 | 30·571 | +3 | + |
| 20-Methylcholanthrene (2 mg/ml) | 7·0±7·5 | −87·6 | 18·155 | +3+ | + |
| Methylene bis(2-chloroaniline) | 31·5±13·7 | −44·2 | 4·966 | +1 | + |
| 2-Methylindole | 58·2±17·6 | +3·0 | 0·296 | −0 | − |
| MNNG | 27·9±17·0 | −50·6 | 4·902 | +2 | + |
| 3-Methyl-4-nitroquinoline-N-oxide | 74·6±7·8 | +32·0 | 6·418 | −0 | − |
| Mitomycin C (2 mg/ml) | 13·6±9·9 | −75·9 | 12·550 | +3+ | + |
| Morgan's base | 17·0±11·3 | −69·9 | 10·308 | +2 | + |
| Naphthalene | 50·9±14·6 | −9·9 | 1·163 | −0 | − |
| 1-Naphthol | 66·1±11·6 | +17·0 | 2·449 | −0 | − |
| 2-Naphthol | 60·3±9·7 | +6·7 | 1·135 | −0 | − |
| 1-Naphthylamine | 51·7±9·9 | −8·5 | 1·403 | −0 | − |
| 2-Naphthylamine | 24·6±8·2 | −56·5 | 10·920 | +2 | + |
| 2-Naphthylamine disulphonic acid | 53·3±10·4 | −5·7 | 0·898 | −0 | − |
| Nitrobenzene | 54·8±15·7 | −3·0 | 0·330 | −0 | − |
| 2-Nitrobiphenyl | 71·3±8·4 | +26·2 | 4·950 | −0 | + |
| 4-Nitrobiphenyl | 42·0±15·9 | −25·7 | 2·641 | +1 | + |
| 2-Nitrofluorene | 72·0±8·9 | +27·4 | 1·449 | −0 | + |
| N-Nitrosodiphenylamine | 40·7±16·3 | −28·0 | 2·960 | +1 | − |
| N-Nitrosoephedrine | 41·6±11·5 | −26·4 | 3·834 | +1 | + |
| N-Nitrosofolic acid | 48·7±10·9 | −13·8 | 1·796 | −0 | + |
| 4-Nitroquinoline-N-oxide | 1·0 (estim) | −98·2 | 1·96 | +3 | + |
| 4-Nonylphenol/ethylene oxide condensate | 52·2±9·7 | −7·6 | 1·22 | −0 | − |
| Orotic acid | 35·0±8·8 | −38·1 | 6·934 | +1 | − |
| Perylene | 69·0±13·4 | +22·1 | 2·798 | −0 | − |
| Phenobarbital | 38·2±16·9 | −32·4 | 3·299 | +1 | − |
| N-phenyl-2-naphthylamine | 45·1±17·8 | −20·2 | 1·972 | +1 | − |
| Propanesultone | 55·3±12·9 | −2·1 | 0·278 | −0 | + |
| β-Propiolactone | 44·8±14·4 | −20·7 | 2·459 | +1 | + |
| Resorcinol | 54·2±7·3 | −4·1 | 0·585 | −0 | − |
| Riboflavin | 45·0±19·9 | −20·4 | 1·785 | −0 | − |
| Safrole | 32·8±12·3 | −41·9 | 5·747 | +1 | + |
| 3,3′,5,5′-Tetramethylbenzidine | 67·1±11·9 | +18·8 | 2·652 | −0 | − |
| Toluene | 60·7±15·1 | +7·4 | 0·845 | −0 | − |
| Toluene-2,4-diisocyanate | 63·1±9·9 | +11·7 | 1·933 | −0 | − |
| 2,4,5-Trichlorophenoxyacetate | 42·1±15·0 | −25·5 | 2·916 | +1 | − |
| Trimethylphosphate | 60·0±14·0 | +6·2 | 0·756 | −0 | − |
| Urethane | 51·2±11·6 | −9·4 | 1·354 | −0 | + |
| Vinyl chloride (61 mg/ml) | 42·6±16·5 | −24·6 | 2·571 | +1 | + |
| Controls (138 samples) | 56·5±16·1 | 0 | | | 0 |

ratio of glands to follicles appeared to vary between one compound and the next, such that the suppression potency of the test compound could be ranked between 0 (no significant effect) and 3 (more than 75% of the glands destroyed). Even so, out of 29 compounds causing more than 50% suppression (*i.e.* grades 2 and 3), 6 were false positives. These compounds were colchicine, dimethylquinoxalene 1,1′-dimethyl-4,4′-bipyridinium dichloride, hydrocortisone, indole and 1,1′-ethylene-2,2′-bipyridinium dibromide.

When the dose-response curves of a limited number of compounds were investigated, it was seen that the system could be made quantitative, in that the dose equivalents of compounds causing 50% gland suppression could be calculated, compared with each other, and related to the activity of benzpyrene. It is, however, debatable whether or not this suppression index has any real value in terms of predicting carcinogenic potency since it could be seen that known potent carcinogens such as aflatoxin appeared to be less active than other potent carcinogens such as benzpyrene. It could be speculated however that the relative carcinogenic potencies of similar chemicals, *e.g.* polycyclic aromatics, could be compared using their respective suppression indices, on the assumption that the mechanism of sebaceous-gland suppression in each case would be similar. Clearly, it would not, however, be valid to compare chemicals of different classes for potency using this technique.

### REFERENCES

BOCK, F. G. & MUND, R. (1958) A Survey of Compounds for Activity in the Suppression of Mouse Sebaceous Glands. *Cancer Res.*, **18**, 892.

CHOUROULINKOV, I., LAZAR, P., IZARD, C., LIBERMAN, C. & GUERIN, M. (1969) Sebaceous Glands and Hyperplasia Tests as Screening Methods for Tobacco Tar Carcinogens. *J. natn. Cancer Inst.*, **42**, 981.

HEALY, P., MAWDSLEY-THOMAS, L. E. & BARRY, D. H. (1970) Short Term Test for Evaluating Potential Carcinogenic Activity of Tobacco Condensates. *Nature*, **228**, 1006.

HEALY, P., MAWDSLEY-THOMAS, L. E. & BARRY, D. H. (1971) The Effect of some Polycyclic Hydrocarbons and Tobacco Condensates on Non-specific Esterase Activity in Sebaceous Glands of Mouse Strain. *J. Path.*, **105**, 147.

SUNTZEFF, V., CARRUTHERS, C. & COWDRY, E. V. (1947) The Role of Sebaceous Glands and Hair Follicles in Epidermal Carcinogenesis. *Cancer Res.*, **7**, 439.

SUNTZEFF, V., COWDRY, E. V. & CRONINGER, A. B. (1955) Microscopic Visualization of the Degeneration of Sebaceous Glands Caused by Carcinogens. *Cancer Res.*, **15**, 637.

SUNTZEFF, V., CRONINGER, A. B., WYNDER, E. L., COWDRY, E. V. & GRAHAM, E. A. (1957) Use of Sebaceous Gland Test of Primary Cigarette Tar Fractions and of Certain Non-carcinogenic Polycyclic Hydrocarbons. *Cancer*, **10**, 250.

TAKIZAWA, H., HOZUMI, M., SUGIMURA, T. & BRYAN, G. T. (1975) Correlation between the Carcinogenicities of Nitrofuran Derivatives and their Destructive Actions in Sebaceous Glands of Mouse Skin. *J. natn. Cancer Inst.*, **54**, 487.